# Supplementary figures and images for: T-REX17 is a transiently expressed non-coding RNA essential for human endoderm formation
Source: eLife. 2023 Jan 31;12:e83077. doi: 10.7554/eLife.83077 (PMC9889090; doi:10.7554/eLife.83077)

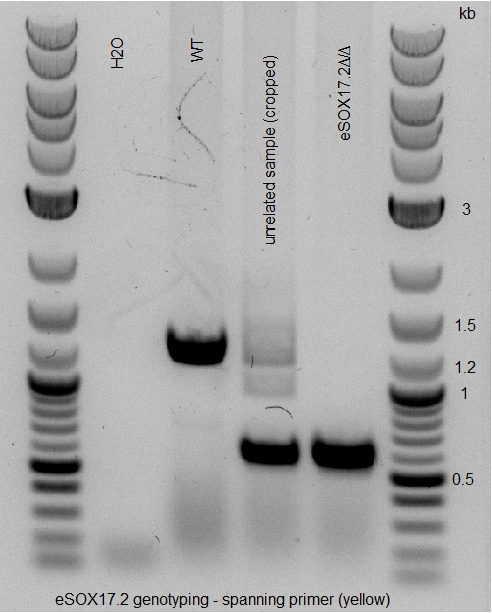

Supplement: Figure 1—figure supplement 1—source data 1. [file elife-83077-fig1-figsupp1-data1.zip › Meissner_30-08-2022-RA-eLife-83077R1_Figure_1_figure_supplement_1_source_data_1/eSOX17.2_genotyping_lower_panel_(yellow, spanning-primer).jpg]

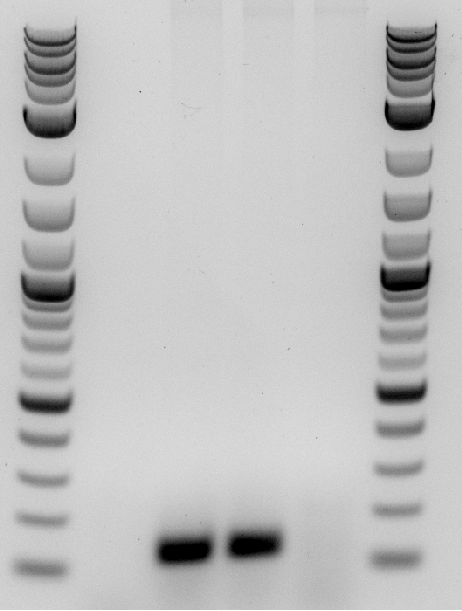

Supplement: Figure 1—figure supplement 1—source data 1. [file elife-83077-fig1-figsupp1-data1.zip › Meissner_30-08-2022-RA-eLife-83077R1_Figure_1_figure_supplement_1_source_data_1/eSOX17.2_genotyping_upper_panel_(blue, internal-primer)_unlabeled.tif]

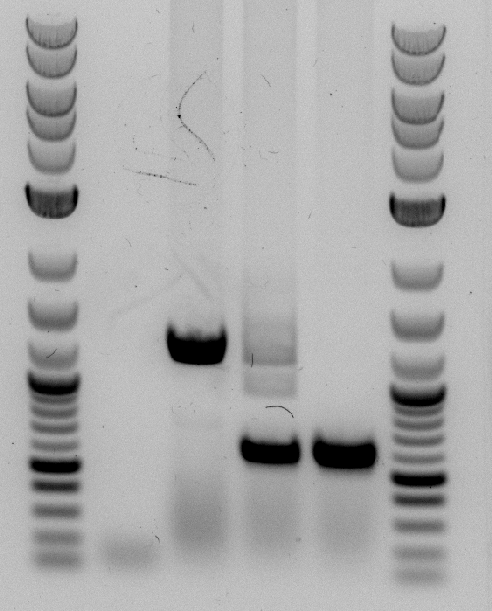

Supplement: Figure 1—figure supplement 1—source data 1. [file elife-83077-fig1-figsupp1-data1.zip › Meissner_30-08-2022-RA-eLife-83077R1_Figure_1_figure_supplement_1_source_data_1/eSOX17.2_genotyping_lower_panel_(yellow, spanning-primer)_unlabeled.tif]

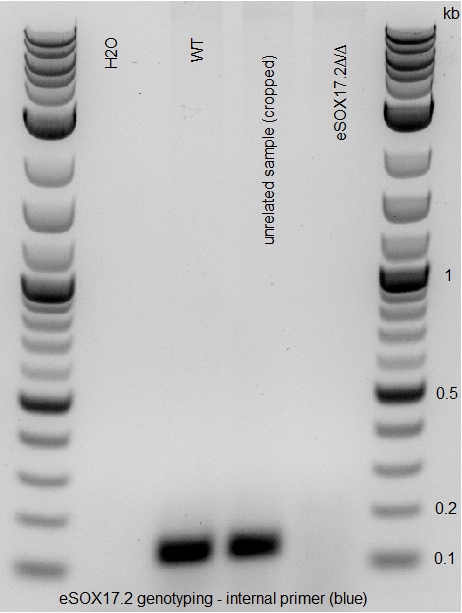

Supplement: Figure 1—figure supplement 1—source data 1. [file elife-83077-fig1-figsupp1-data1.zip › Meissner_30-08-2022-RA-eLife-83077R1_Figure_1_figure_supplement_1_source_data_1/eSOX17.2_genotyping_upper_panel_(blue, internal-primer).jpg]

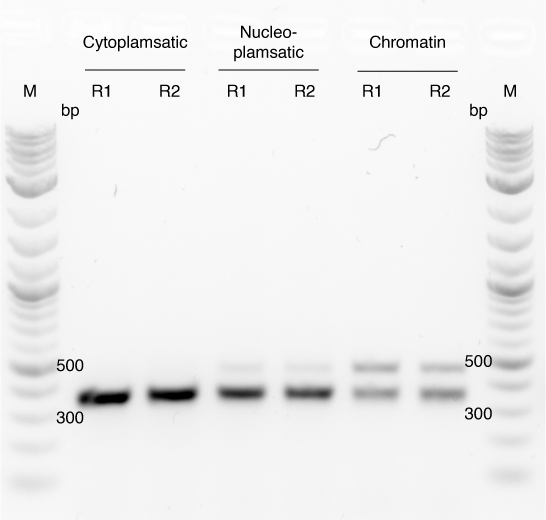

Supplement: Figure 2—figure supplement 1—source data 1. [file elife-83077-fig2-figsupp1-data1.zip › Meissner_30-08-2022-RA-eLife-83077R1_Figure_2_figure_supplement_1_source_data_1/Cell_fractionation_GAPDH_labeled.tif]

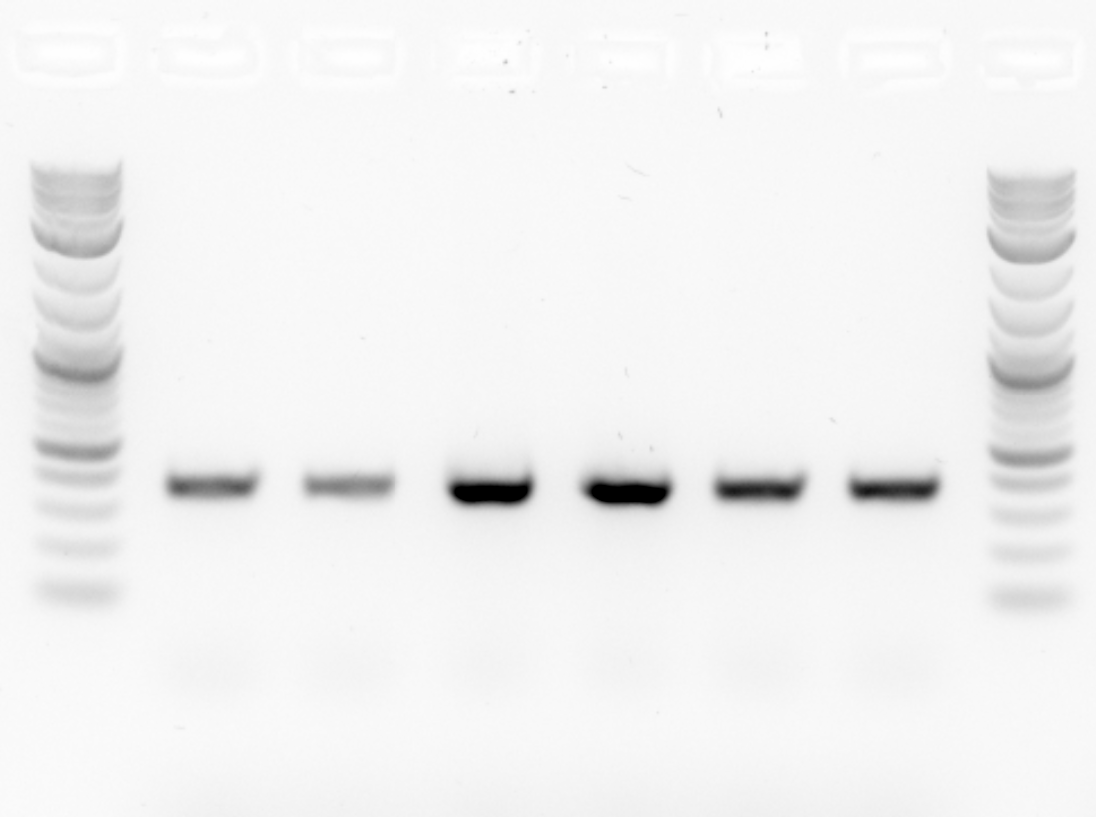

Supplement: Figure 2—figure supplement 1—source data 1. [file elife-83077-fig2-figsupp1-data1.zip › Meissner_30-08-2022-RA-eLife-83077R1_Figure_2_figure_supplement_1_source_data_1/Cell_fractionation_LNCSOX17.tif]

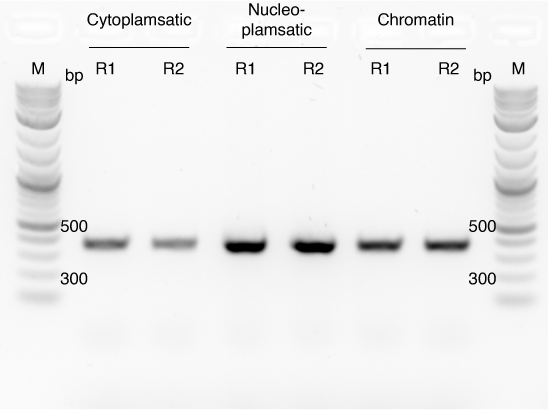

Supplement: Figure 2—figure supplement 1—source data 1. [file elife-83077-fig2-figsupp1-data1.zip › Meissner_30-08-2022-RA-eLife-83077R1_Figure_2_figure_supplement_1_source_data_1/Cell_fractionation_T-REX17_labeled.tif]

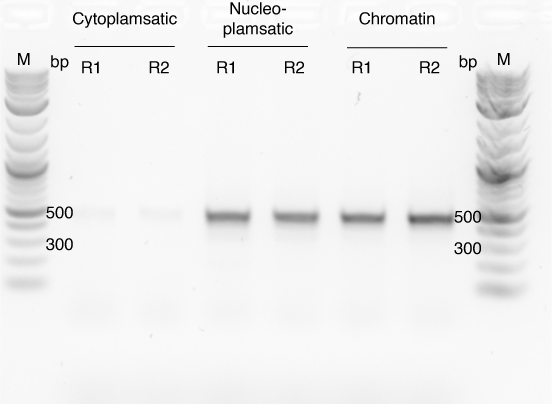

Supplement: Figure 2—figure supplement 1—source data 1. [file elife-83077-fig2-figsupp1-data1.zip › Meissner_30-08-2022-RA-eLife-83077R1_Figure_2_figure_supplement_1_source_data_1/Cell_fractionation_XIST_labeled.tif]

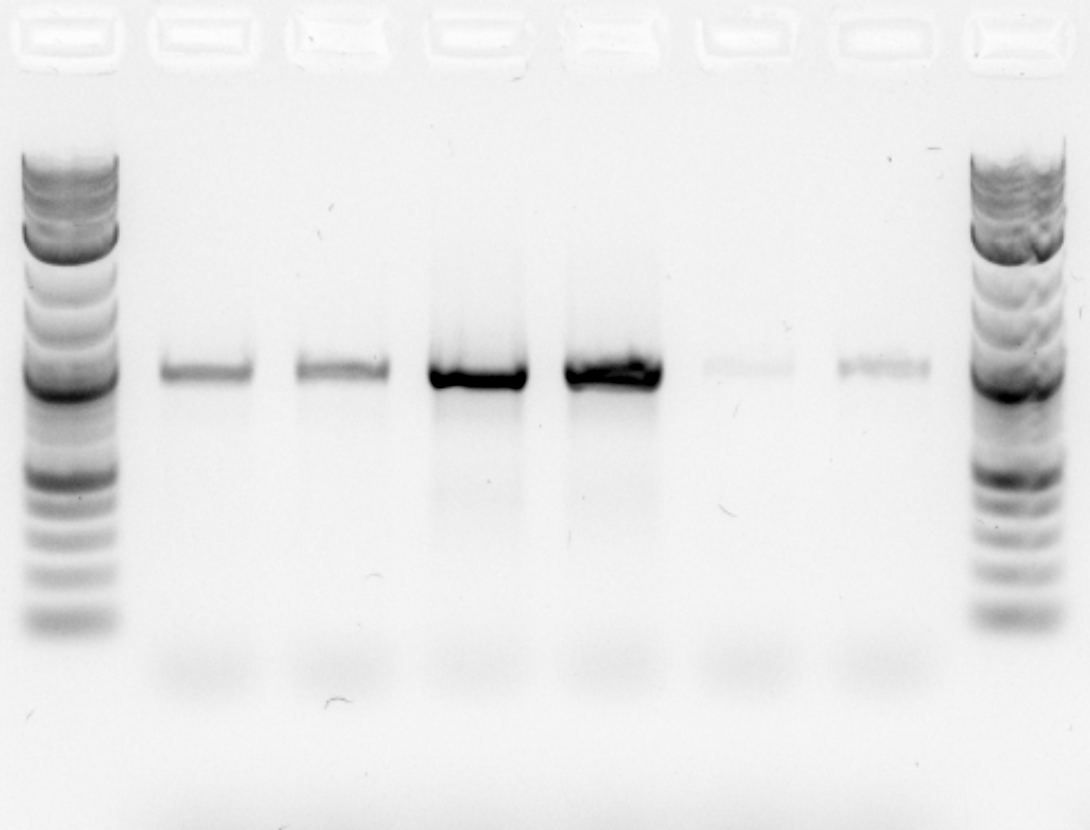

Supplement: Figure 2—figure supplement 1—source data 1. [file elife-83077-fig2-figsupp1-data1.zip › Meissner_30-08-2022-RA-eLife-83077R1_Figure_2_figure_supplement_1_source_data_1/Cell_fractionation_MALAT1.tif]

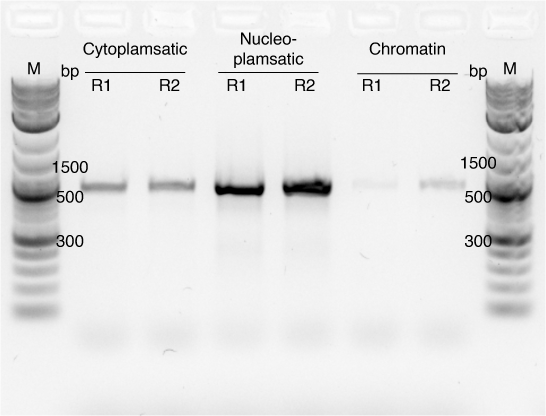

Supplement: Figure 2—figure supplement 1—source data 1. [file elife-83077-fig2-figsupp1-data1.zip › Meissner_30-08-2022-RA-eLife-83077R1_Figure_2_figure_supplement_1_source_data_1/Cell_fractionation_MALAT1_labeled.tif]

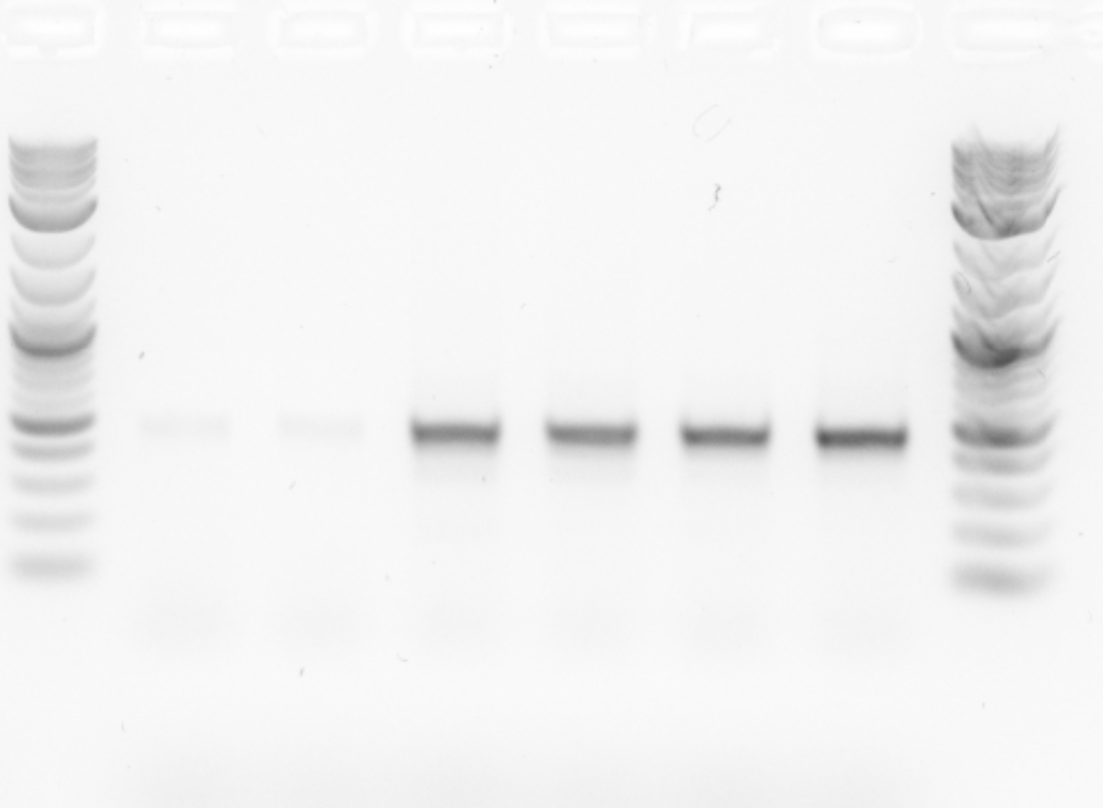

Supplement: Figure 2—figure supplement 1—source data 1. [file elife-83077-fig2-figsupp1-data1.zip › Meissner_30-08-2022-RA-eLife-83077R1_Figure_2_figure_supplement_1_source_data_1/Cell_fractionation_XIST.tif]

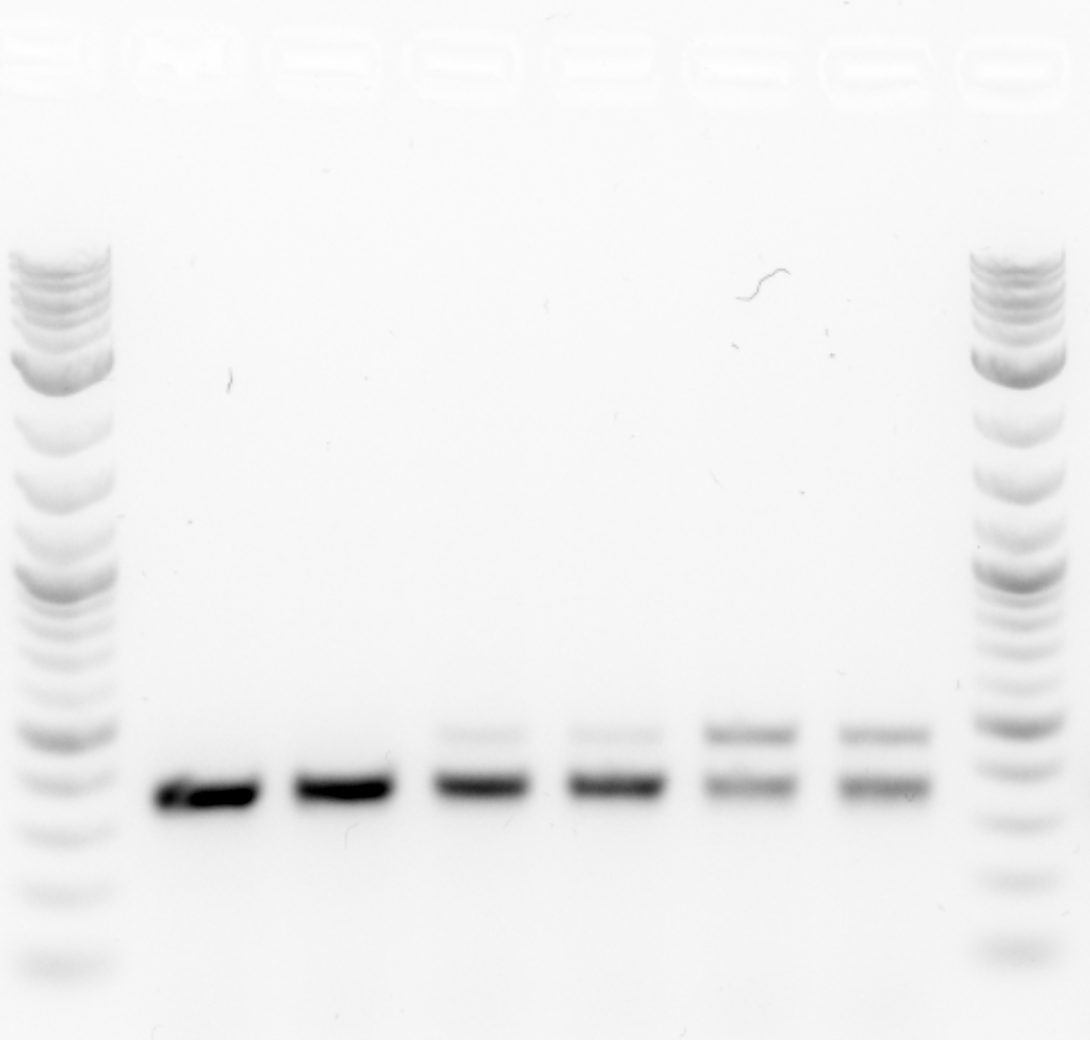

Supplement: Figure 2—figure supplement 1—source data 1. [file elife-83077-fig2-figsupp1-data1.zip › Meissner_30-08-2022-RA-eLife-83077R1_Figure_2_figure_supplement_1_source_data_1/Cell_fractionation_GAPDH.tif]

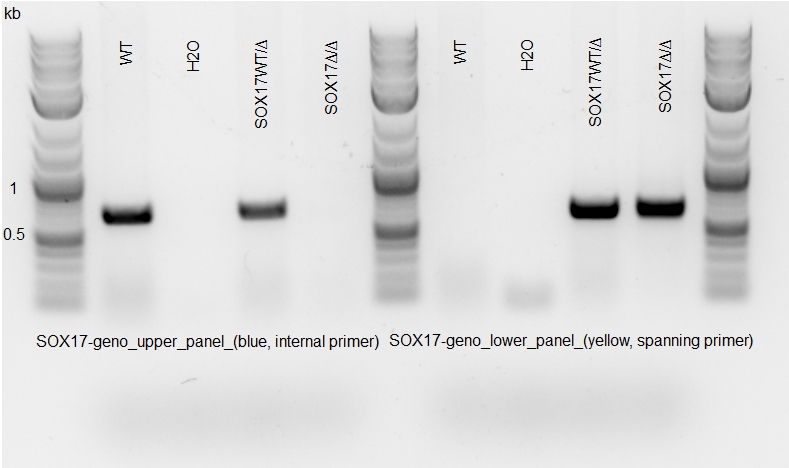

Supplement: Figure 3—figure supplement 1—source data 1. [file elife-83077-fig3-figsupp1-data1.zip › Meissner_30-08-2022-RA-eLife-83077R1_Figure_3_figure_supplement_1_source_data_1 (1)/SOX17-KO_genotyping_labeled.jpg]

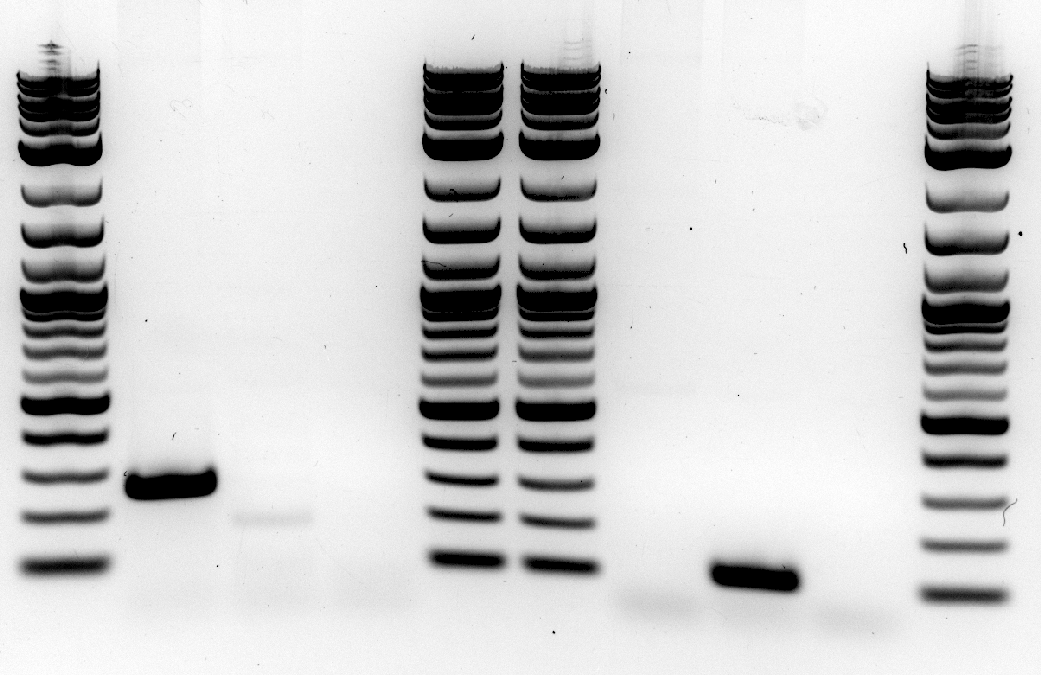

Supplement: Figure 3—figure supplement 1—source data 1. [file elife-83077-fig3-figsupp1-data1.zip › Meissner_30-08-2022-RA-eLife-83077R1_Figure_3_figure_supplement_1_source_data_1 (1)/ePoly(A)-KI_genotyping_unlabeled.tif]

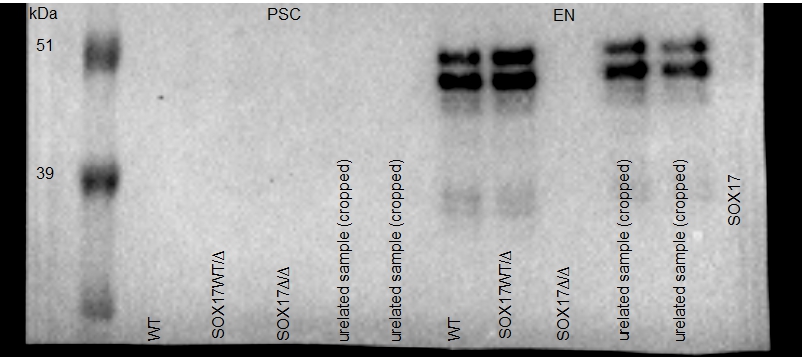

Supplement: Figure 3—figure supplement 1—source data 1. [file elife-83077-fig3-figsupp1-data1.zip › Meissner_30-08-2022-RA-eLife-83077R1_Figure_3_figure_supplement_1_source_data_1 (1)/Day_0_(PSC, left)_+Day_5_(EN, right)_SOX17-WB_labeled.jpg]

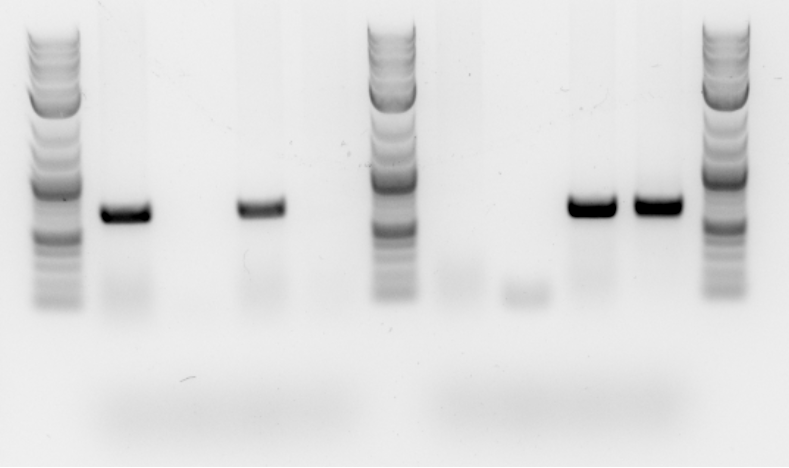

Supplement: Figure 3—figure supplement 1—source data 1. [file elife-83077-fig3-figsupp1-data1.zip › Meissner_30-08-2022-RA-eLife-83077R1_Figure_3_figure_supplement_1_source_data_1 (1)/SOX17-KO_genotyping_unlabeled.tif]

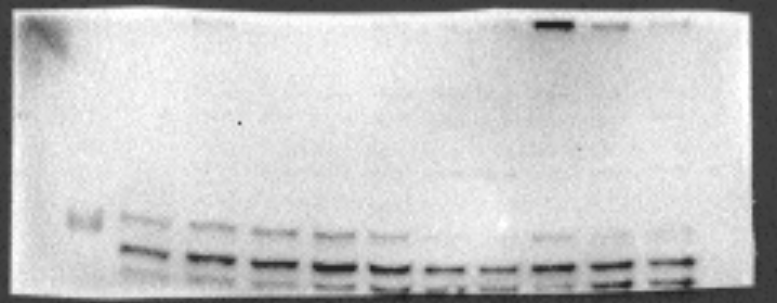

Supplement: Figure 3—figure supplement 1—source data 1. [file elife-83077-fig3-figsupp1-data1.zip › Meissner_30-08-2022-RA-eLife-83077R1_Figure_3_figure_supplement_1_source_data_1 (1)/Day_0_(PSC, left)_+Day_5_(EN, right)_LAMIN-B-WB_unlabeled.tif]

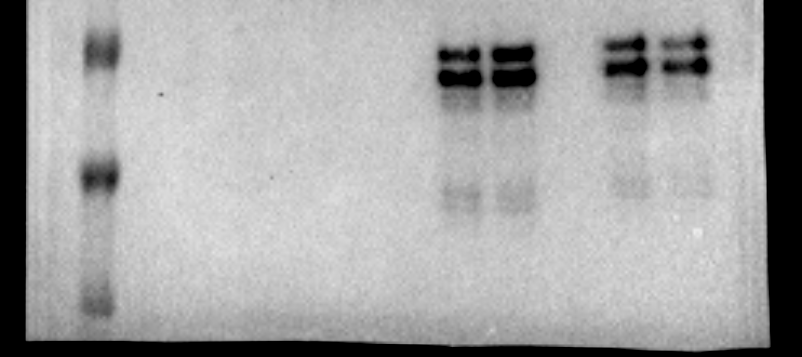

Supplement: Figure 3—figure supplement 1—source data 1. [file elife-83077-fig3-figsupp1-data1.zip › Meissner_30-08-2022-RA-eLife-83077R1_Figure_3_figure_supplement_1_source_data_1 (1)/Day_0_(PSC, left)_+Day_5_(EN, right)_SOX17-WB_unlabeled.tif]

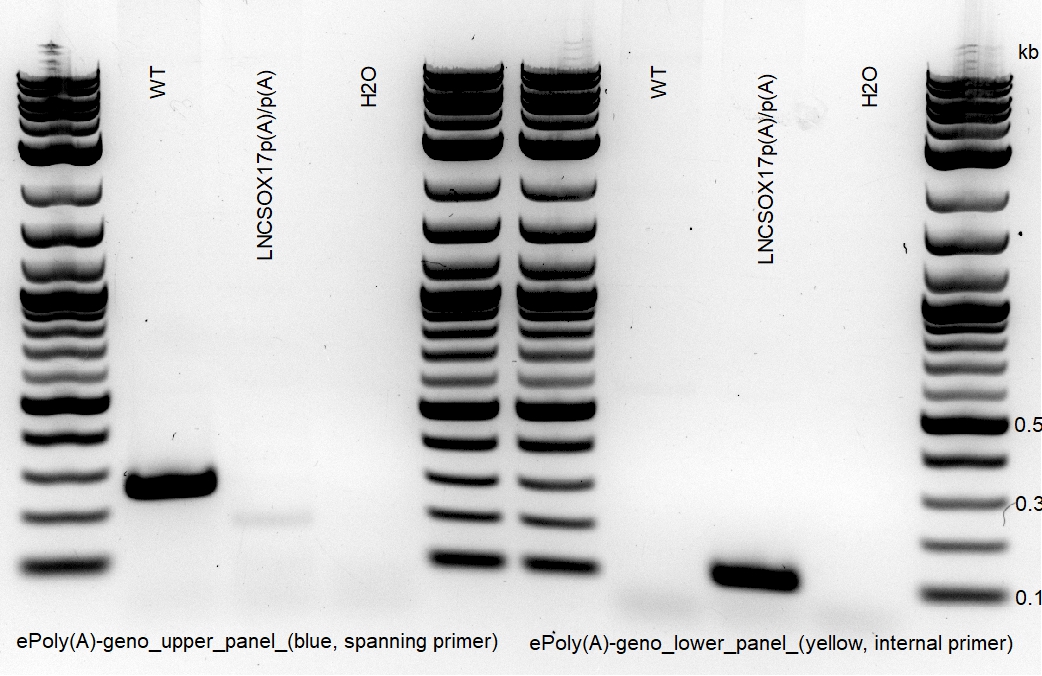

Supplement: Figure 3—figure supplement 1—source data 1. [file elife-83077-fig3-figsupp1-data1.zip › Meissner_30-08-2022-RA-eLife-83077R1_Figure_3_figure_supplement_1_source_data_1 (1)/ePoly(A)-KI_genotyping_labeled.jpg]

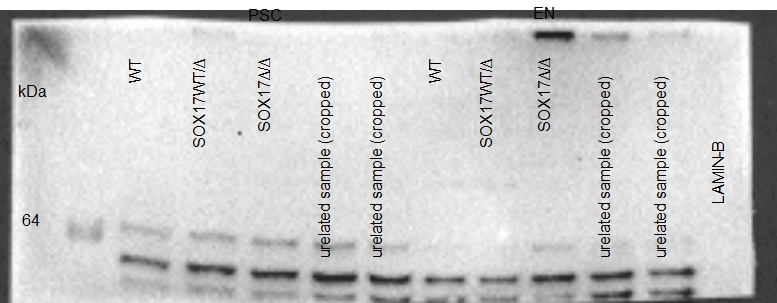

Supplement: Figure 3—figure supplement 1—source data 1. [file elife-83077-fig3-figsupp1-data1.zip › Meissner_30-08-2022-RA-eLife-83077R1_Figure_3_figure_supplement_1_source_data_1 (1)/Day_0_(PSC, left)_+Day_5_(EN, right)_LAMIN-B-WB_labeled.jpg]

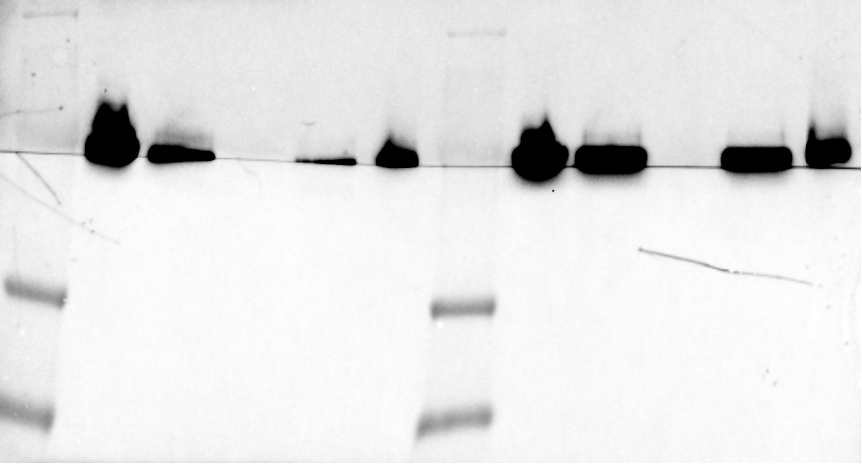

Supplement: Figure 3—figure supplement 2—source data 1. [file elife-83077-fig3-figsupp2-data1.zip › Meissner_30-08-2022-RA-eLife-83077R1_Figure_3_figure_supplement_2_source_data_1 (1)/hnRNPU_RIP_WB_unlabeled.tif]

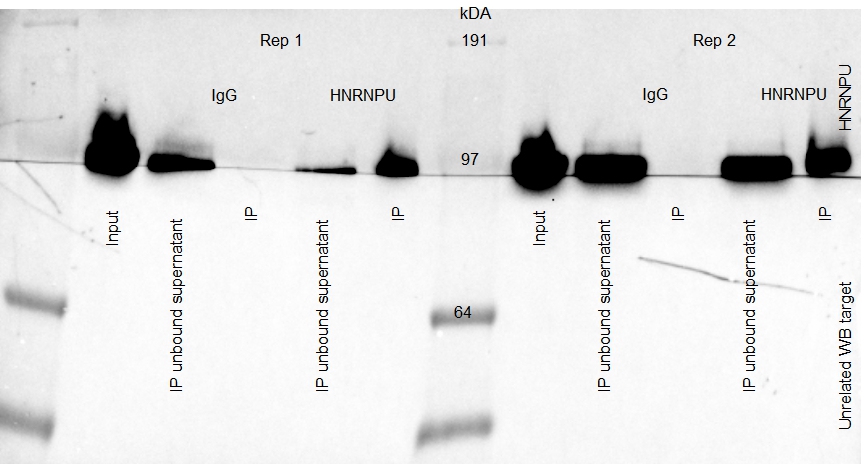

Supplement: Figure 3—figure supplement 2—source data 1. [file elife-83077-fig3-figsupp2-data1.zip › Meissner_30-08-2022-RA-eLife-83077R1_Figure_3_figure_supplement_2_source_data_1 (1)/hnRNPU_RIP_WB_labeled.jpg]

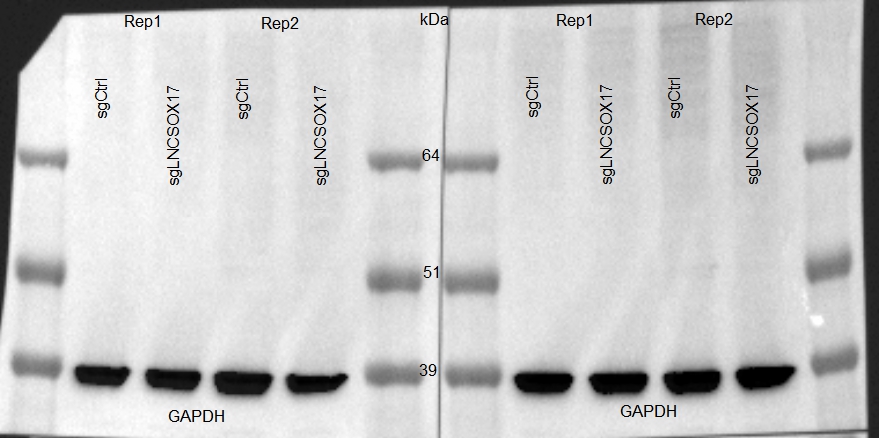

Supplement: Figure 4—source data 1. [file elife-83077-fig4-data1.zip › Figure 4 - source data 1 83077/Day_5_GAPDH(left)_GAPDH(right)_raw_labeled.jpg]

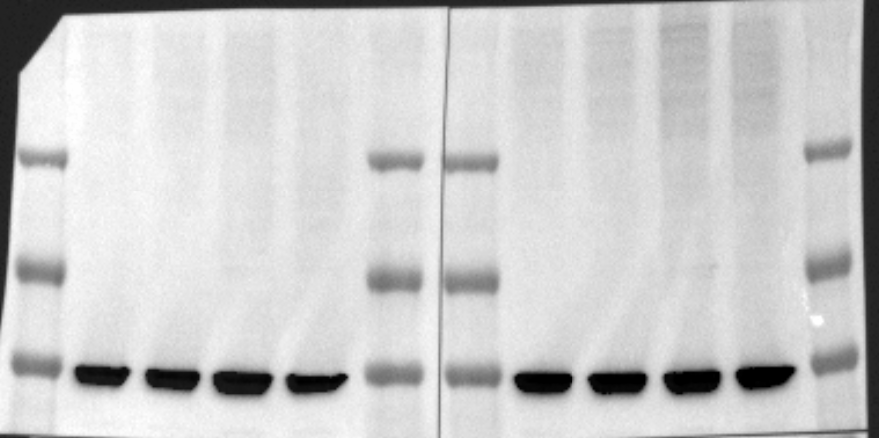

Supplement: Figure 4—source data 1. [file elife-83077-fig4-data1.zip › Figure 4 - source data 1 83077/Day_5_GAPDH(left)_GAPDH(right)_raw_unlabeled.tif]

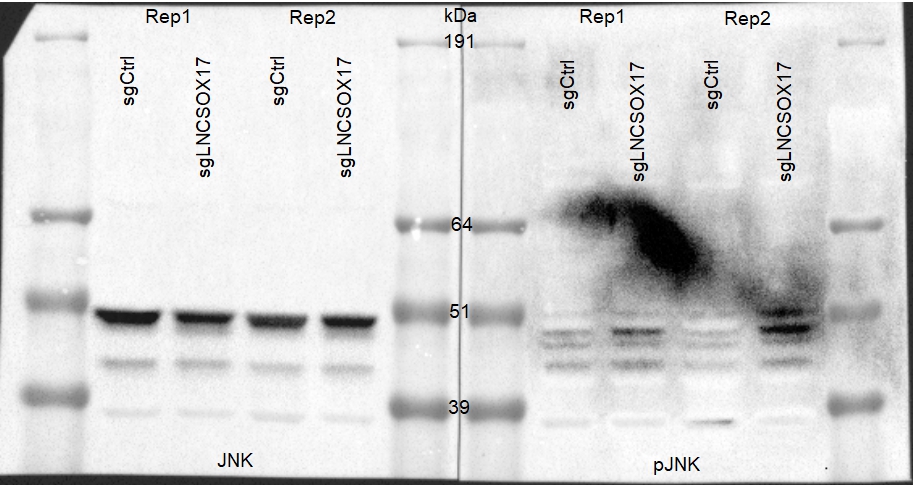

Supplement: Figure 4—source data 1. [file elife-83077-fig4-data1.zip › Figure 4 - source data 1 83077/Day_5_JNK(left)_pJNK(right)_raw_labeled.jpg]

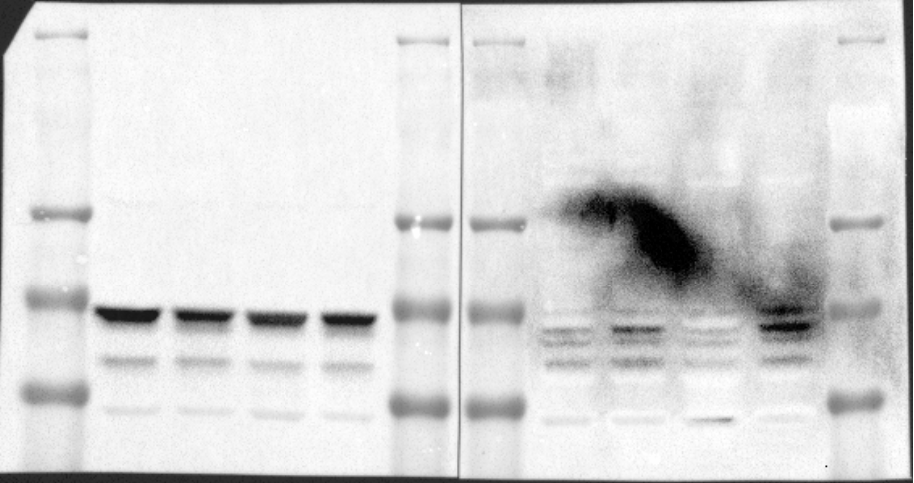

Supplement: Figure 4—source data 1. [file elife-83077-fig4-data1.zip › Figure 4 - source data 1 83077/Day_5_JNK(left)_pJNK(right)_raw_unlabeled.tif]

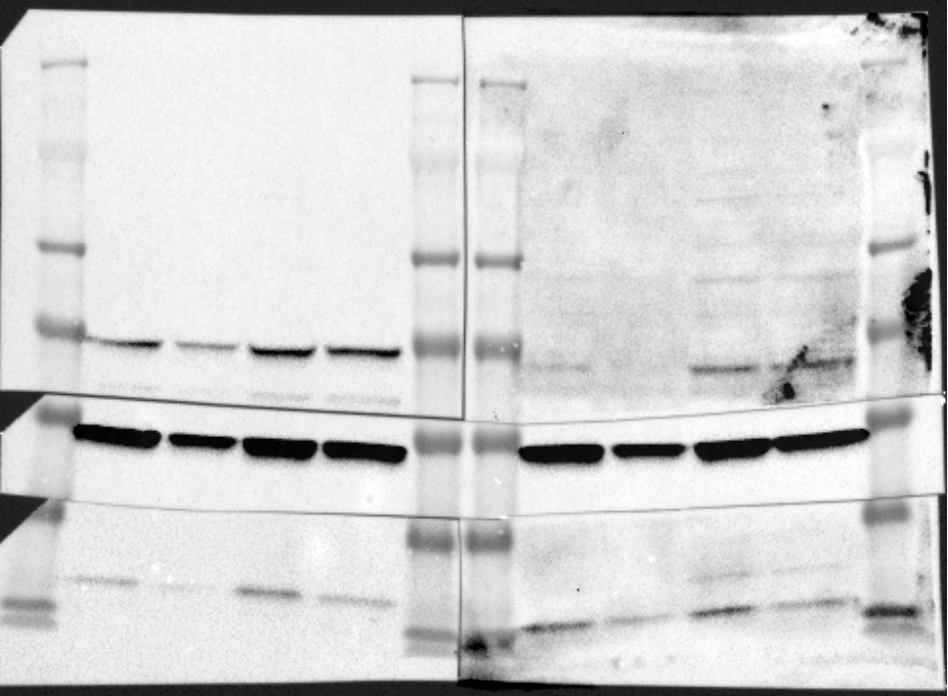

Supplement: Figure 4—figure supplement 2—source data 1. [file elife-83077-fig4-figsupp2-data1.zip › Meissner_30-08-2022-RA-eLife-83077R1_Figure_4_figure_supplement_2_source_data_1/Day_0_JNK(left)_pJNK(right)_WB_raw_unlabeled.tif]

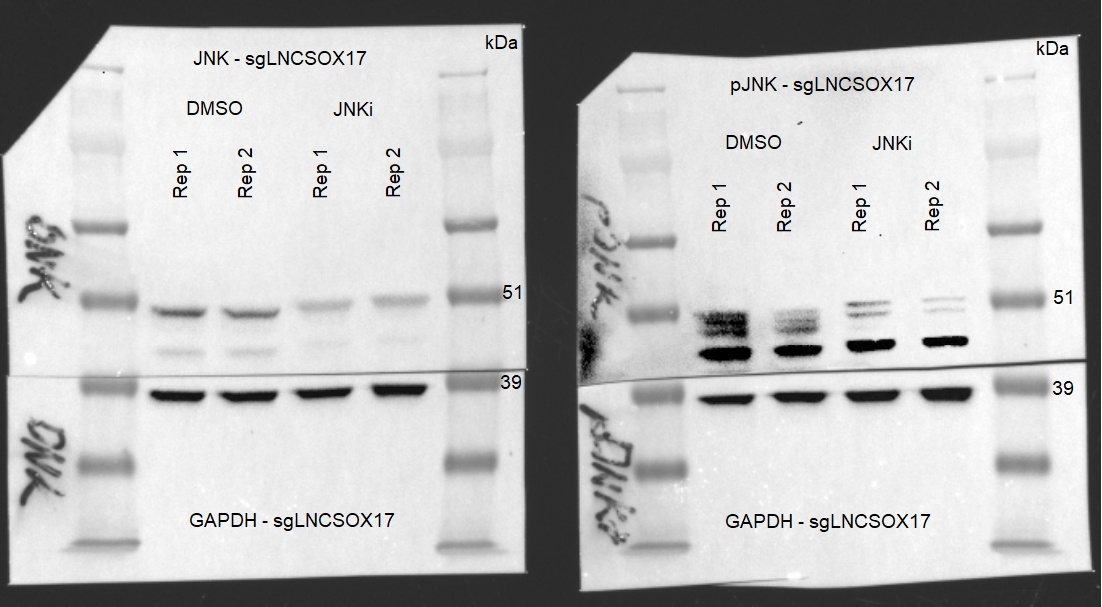

Supplement: Figure 4—figure supplement 2—source data 1. [file elife-83077-fig4-figsupp2-data1.zip › Meissner_30-08-2022-RA-eLife-83077R1_Figure_4_figure_supplement_2_source_data_1/JNKi_WB_Day_5_JNK(left)_pJNK(right)_raw_labeled.jpg]

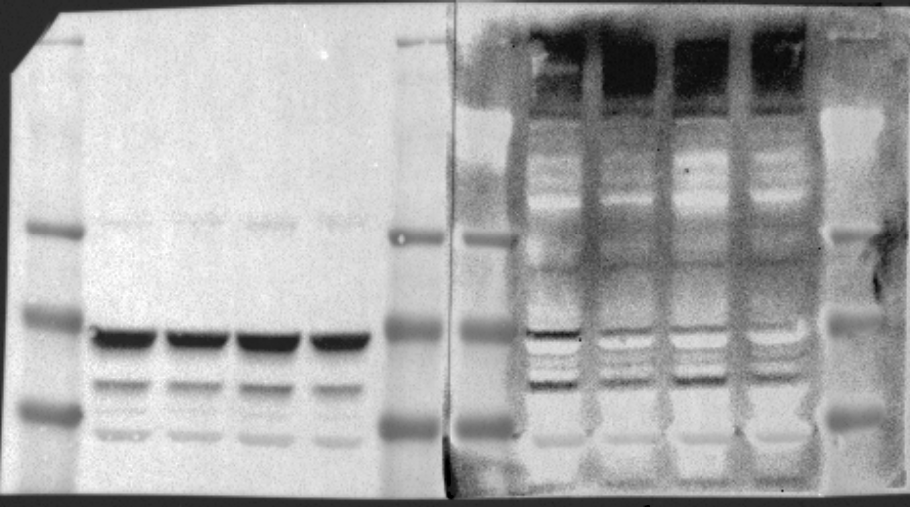

Supplement: Figure 4—figure supplement 2—source data 1. [file elife-83077-fig4-figsupp2-data1.zip › Meissner_30-08-2022-RA-eLife-83077R1_Figure_4_figure_supplement_2_source_data_1/Day_3_JNK(left)_pJNK(right)_WB_raw_unlabeled.tif]

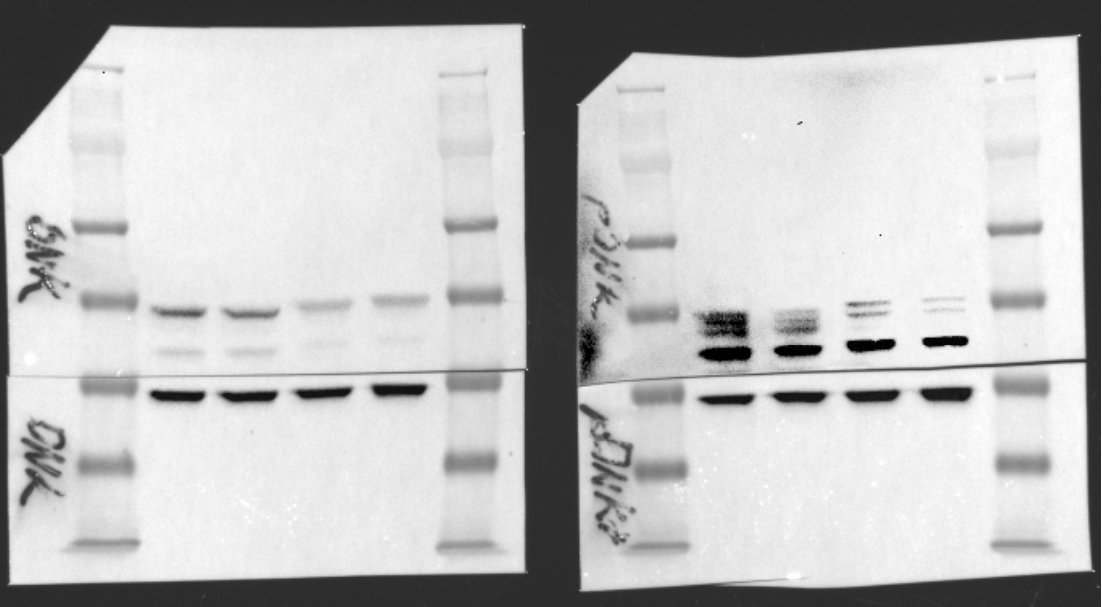

Supplement: Figure 4—figure supplement 2—source data 1. [file elife-83077-fig4-figsupp2-data1.zip › Meissner_30-08-2022-RA-eLife-83077R1_Figure_4_figure_supplement_2_source_data_1/JNKi_WB_Day_5_JNK(left)_pJNK(right)_raw_unlabeled.tif]

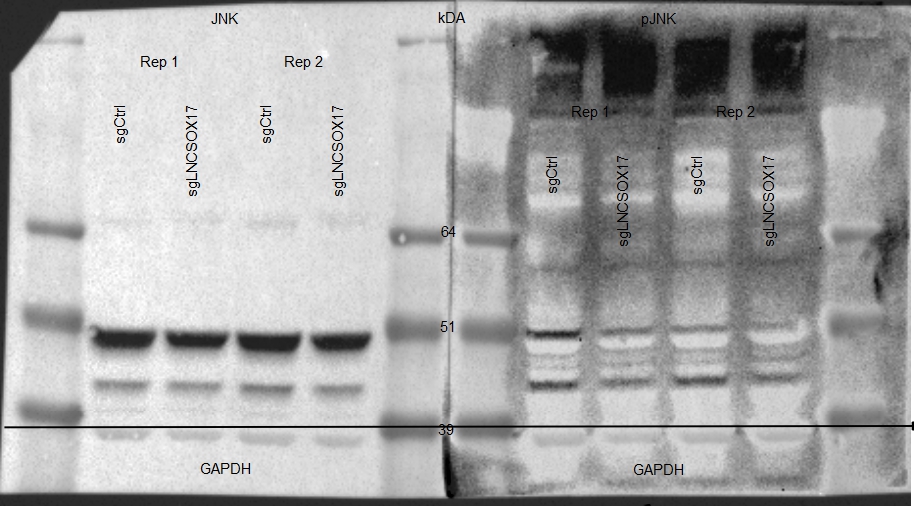

Supplement: Figure 4—figure supplement 2—source data 1. [file elife-83077-fig4-figsupp2-data1.zip › Meissner_30-08-2022-RA-eLife-83077R1_Figure_4_figure_supplement_2_source_data_1/Day_3_JNK(left)_pJNK(right)_WB_raw_labeled.jpg]

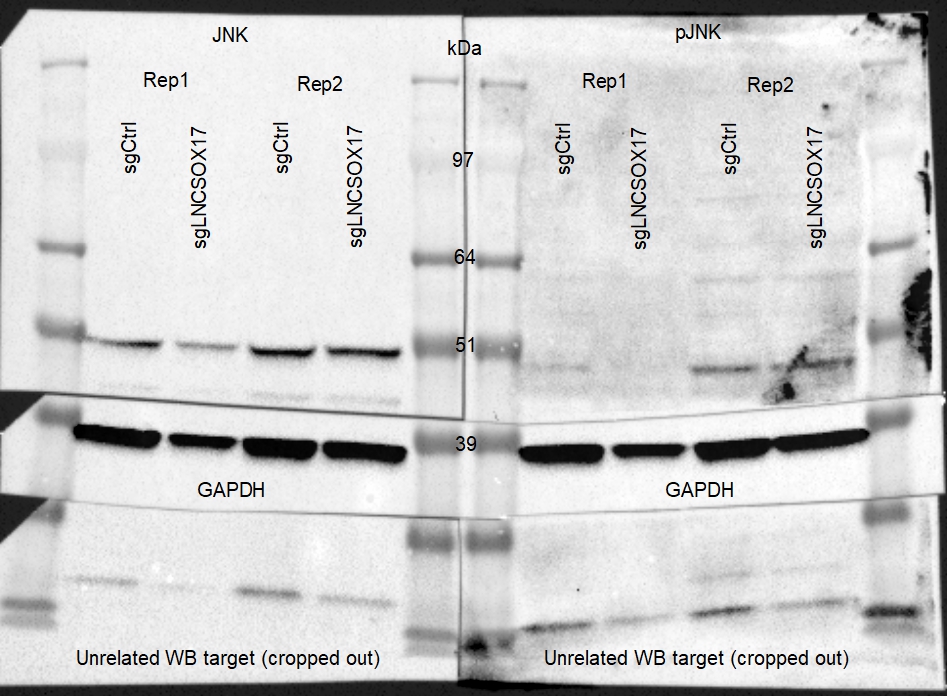

Supplement: Figure 4—figure supplement 2—source data 1. [file elife-83077-fig4-figsupp2-data1.zip › Meissner_30-08-2022-RA-eLife-83077R1_Figure_4_figure_supplement_2_source_data_1/Day_0_JNK(left)_pJNK(right)_WB_raw_labeled.jpg]
